# Physiological and Anatomical Responses of Faba Bean Plants Infected with Chocolate Spot Disease to Chemical Inducers

**DOI:** 10.3390/life13020392

**Published:** 2023-01-31

**Authors:** Rasha M. Alnefaie, Sahar A. EL-Sayed, Amany A. Ramadan, Ahmed I. Elmezien, Ahmed M. El-Taher, Timothy O. Randhir, Ahmed Bondok

**Affiliations:** 1 Biology Department, College of Science, Albaha University, Al Bahah 65779, Saudi Arabia; 2 Institute of Plant Pathology, Agricultural Research Center, Giza P.O. Box 12613, Egypt; 3 Botany Department, Agricultural and Biological Research Institute, National Research Centre, Dokki, Giza P.O. Box 12622, Egypt; 4 Department of Agricultural Botany (Plant Physiology), Faculty of Agriculture, Al-Azhar University, Cairo 11884, Egypt; 5 Department of Agricultural Botany, Faculty of Agriculture, Al-Azhar University, Cairo 11884, Egypt; 6 Department of Environmental Conservation, University of Massachusetts, Amherst, MA 01003, USA; 7 Department of Plant Pathology, Faculty of Agriculture, Ain Shams University, Cairo 11566, Egypt

**Keywords:** chocolate spot disease, *Botrytis fabae*, faba bean, antioxidant enzymes, protein banding and anatomy

## Abstract

Plant diseases are biotic stresses that restrict crop plants’ ability to develop and produce. Numerous foliar diseases, such as chocolate spots, can cause significant production losses in *Vicia faba* plants. Certain chemical inducers, including salicylic acid (SA), oxalic acid (OA), nicotinic acid (NA), and benzoic acid (BA), were used in this study to assess efficacy in controlling these diseases. A foliar spray of these phenolic acids was used to manage the impacts of the biotic stress resulting from disease incidence. All tested chemical inducers resulted in a significant decrease in disease severity. They also enhanced the defense system of treated plants through increasing antioxidant enzyme activity (Peroxidase, polyphenol oxidase, β-1, 3-glucanase, and chitinase) compared to the corresponding control. Healthy leaves of faba plants recorded the lowest (*p* < 0.05) values of all antioxidant activities compared to those plants infected by *Botrytis fabae*. Moreover, the separation of proteins using SDS-PAGE showed slight differences among treatments. Furthermore, foliar spray with natural organic acids reduced the adverse effects of fungal infection by expediting recovery. The SA (5 mM) treatment produced a pronounced increase in the upper, lower epidermis, palisade thickness, spongy tissues, midrib zone, length, and width of vascular bundle. The foliar application with other treatments resulted in a slight increase in the thickness of the examined layers, especially by benzoic acid. In general, all tested chemical inducers could alleviate the adverse effects of the biotic stress on faba bean plants infected by *Botrytis fabae*.

## 1. Introduction

Faba bean (*Vicia faba*) is Egypt’s most important legume crop and is widely produced throughout the Mediterranean as a protein source for human and animal consumption [1]. The high protein content of faba bean ranges from 25 to 35 percent and has been credited with its nutritional value. The seeds are also high in thiamin, tocopherols, niacin, and folic acid and are notably high in calcium and iron [2]. At the same time, its cultivation increases the amount of nitrogen in the soil [3].

Chocolate spot disease of faba bean is caused by *Botrytis fabae* and *B. cinerea* and is considered the most important disease in the Northern region of the Egyptian Nile Delta [4], which has relatively low temperature and, at the same time, high relative humidity that favor the spread of this disease [5]. The disease causes a severe yield loss reaching 60–80% among the susceptible cultivars [6]. It decreases the total carbohydrates, nitrogen, nucleic acid, and protein contents of the yielded seeds [7]. Under stress conditions, the production of reactive oxygen species (ROS) (known for signaling intermediates during abiotic and biotic stress conditions) increases and causes plant oxidative stress [8]. In fact, ROS damages cellular membranes in the processes of lipid peroxidation and are also able to cause harmful effects on DNA, proteins, and chlorophyll [9]. Plants produce several major antioxidant enzymes, for example, superoxide dismutase (SOD), which has an essential role in singlet oxygen and scavenging ROS from the cytosol, mitochondria, and chloroplasts in the cell [10].

Utilization of chemical inducers is a new approach in fungal and bacterial infections control within an environmentally friendly defense system in crop plants. These substances induce resistance throughout the signal transduction system, which promotes the production of specific enzymes that catalyze biosynthetic responses to form resistance compounds such as polyphenols, and pathogenesis-related proteins that enhance the plant resistance to pathogens [11]. Salicylic acid (SA) is an endogenous growth regulator that works as a phenolic non-enzymatic antioxidant (a defense mechanism in plants against stress conditions) that helps plants to regulate some physiological activities [12]. The SA also plays a vital role in the plant growth and development, seed germination, pigmentation, photosynthesis, transpiration rate, ion uptake and transport, fruit yield, glycolysis, and induces changes in leaf anatomy and chloroplast ultrastructure [13]. The use of SA significantly reduced chocolate spot disease severity in faba beans caused by *B. fabae* [14,15].

From the anatomical point of view, Cárcamo et al. [16] on *Zea mays*, L., Nour et al. [17] on bean and Gomaa et al. [18] on *Lupinus termis* L., reported that SA minimized the harmful effects of stress conditions. Applying SA improved anatomical measurements of cell wall, epidermis, fiber strands, cortex, xylem and phloem tissues, the parenchymatous area of the pith and vessel diameter, midvein, and leaflet lamina. Benzoic acid (BA) is a natural antioxidant organic acid also considered a biosynthetic precursor of SA [19]. It works as a key intermediate in shikimate and phenyl propanoid pathways. Shikimic acid is a precursor of many alkaloids, aromatic amino acids, and indole derivatives that improve plant growth and productivity [20] and provide plants with abiotic stress tolerance [21].

Moreover, oxalic acid (OA) is crucial in controlling fungal infection [22] since fungal mutants deficient in OA production were non-pathogenic to common bean plants [23]. Decreasing OA accumulation by using fungal mutants or the overexpression of oxalate oxidase leads to ROS generation, allowing the plant to activate some defense responses [24]. At later stages of pathogen infection, OA reduces ROS production [25]. As a result, at an advanced stage of *Sclerotinia sclerotiorum*, the plant antioxidant system most likely plays a role in inhibiting ROS formation [26]. Nicotinic acid (NA), known also as niacin, nicotinamide, and vitamin B_3_, is a known component of the pyridine dinucleotide coenzymes NADH and NADPH, which are involved in a variety of enzymatic oxidation-reduction events in plant cells [27]. Nicotinamide is a growth-regulating substance that can modify various physiological features of plants in small amounts [28]. Moreover, nicotinamide is a stress-related chemical that causes and controls the activity of the secondary metabolic accumulation process and/or defensive metabolism expressed in plants [29]. Niacin may be utilized to improve stress tolerance in kiwi fruit when exposed to short-term stressful conditions [30]. Furthermore, foliar spray with the niacin solution increased NADPH and NADP^+^ levels and decreased both O_2_^−^ generation and H_2_O_2_ content for a short time.

The present study examines the protective effects of selected organic acids and resistance inducers in controlling the chocolate spot disease of faba bean plants and studying their effects on the antioxidant defense system. 

## 2. Materials and Methods

Chocolate spot disease of faba bean was surveyed at six Egyptian Governorates, namely El-Beheira (Nubaria and Kafr-Eldawar), Kafer El-Sheik (Sakha), Gharbiya (Tanta), Minufiya (Serce-Alian), Sharkia (Zagazig) and Qalubia (Qalub). The severity of the chocolate spot disease of faba bean in Egypt varied by local weather (temperature and humidity) in each Governorate.

The survey was conducted during 2019/2020 and 2020/2021 growing seasons, where the survey was chosen to coincide with the flowering, fruiting, and late fruiting stages of faba bean when the disease reached its peak [31,32]. The survey of the examined sites started at one corner of each field and transected in an M-shaped pattern for approximately 800 paces, stopping at ten equally spaced spots along the way for sampling.

### 2.1. Studies on the Causal Pathogens

#### 2.1.1. Isolation of Chocolate Spot Pathogens

Samples of faba bean leaves naturally infected with chocolate spot disease symptoms were collected from the studied locations at the flowering stage. The infected leaves were cut into small pieces (5 mm), each containing a single lesion. The infected tissues were sterilized by soaking in 5% sodium hypochlorite for two minutes, then washed thoroughly several times with sterilized distilled water and dried between two layers of sterilized filter paper. The surface sterilized pieces were transferred onto potato dextrose agar (PDA) plates at the rate of five pieces/plate. All plates were incubated at 20 ± 1 °C for 5–7 days. The isolated fungi were purified using the hyphal tip technique [33].

#### 2.1.2. Identification of Isolated Fungi

Isolated fungi were identified as described by Moussa et al. [34] according to their morphological and microscopical characteristics. The identification was carried out at the Department of Mycology, Survey and Identification Unit, Plant Pathology Research Institute, Agriculture Research Center, Giza, Egypt. Pure cultures of each isolate were kept on PDA slants at 4 °C for further studies.

#### 2.1.3. Pathogenicity Test

Pathogenicity test was carried out using seven local isolates of *B. fabae*, i.e., (Nubaria, Kafer-Eldawer, Sakha, Tanta, Serce-Alian, Zagazig, and Qalub) under greenhouse conditions.

##### Inoculum Preparation

Isolates of *B. fabae* were grown on leaves of faba bean extract agar media. For spore suspension preparation, the medium was added into sterilized Petri dishes prior to solidification. Then the solidified media in plates were inoculated on equal discs (5 mm) of each test isolate and incubated at a temperature of 20 °C ± 1 for a period of 12 days [35] under alternating light (12 h) and darkness (12 h) procedure in automatically incubated to boost the production of spores. For replication, a total of ten plates were used for each isolate. When the incubation period passed, 10 mL of distilled sterilized water was added to the plates and then brushed carefully using a rubber brush. Three layers of cheesecloth were used to filter the suspension in order to eliminate the residues of mycelia. A Spencer Haemacytometer slide was used to count the number of spores/mL in the spore suspension, and then the spores/mL rate was adjusted to approximately 2.5 × 10^5^ of *B. fabae.*

##### Plant Preparation

Faba bean susceptible cultivar Giza 429 (*Vicia faba*, L.) used in these experiments were obtained from the Field Crops Research Institute, Agricultural Research Center, Giza, Egypt. Four abiotic inducers (SA, NA, OA, and BA) were obtained from Sigma Company.

Faba bean plants were grown in plastic pots (20 cm), each planted with eight seeds and thinned to five plants/pot with five pots specified for each treatments under greenhouse conditions. After forty-five days from sowing, each group of faba bean plants was sprayed until runoff of abiotic inducers 24 h before inoculation with *B. fabae* at the rates of 1, 3, and 5 mM for salicylic acid (SA), 1, 2, and 3 mM for Nicotinic acid (NA) and Oxalic acid (OA). Furthermore, Benzoic acid (BA) was applied at 0.8, 1.6, and 3.2 mM. All examined materials were firstly dissolved in 2 mL of 100% dimethyl sulfoxide (DMSO) and then adjusted to the final concentration using sterilized water for each inducer to examine the possibility of alleviating the adverse effects of chocolate spot. The sprayed plants were covered with polyethylene bags for two days before spraying with *B. fabae* spore suspension (2.5 × 10^5^ spores/mL), about 10 mL/each pot. Whereas the control plants were sprayed with 10 mL sterilized water only.

##### Pathogenicity Assessment and Development of Choloate Spot Disease

The inoculated plants were examined for chocolate spot disease infection. The diease severity were recorded after 2, 3, and 5 days of spray *B. fabae* inoculation. This test was done under greenhouse conditions following Bernier et al. [36]. Moreover, four abiotic inducers, SA, NA, OA, and BA, were used as comparison treatments. Each treatment was repeated three times, and the experimental design was a randomized complete block design under open field conditions to investigate their effectiveness against chocolate spot disease severity in faba bean. The inducers were sprayed twice, first at 15 days (at the 1st leaf stage) while the other was at 30 days (at the 6th leaf) from sowing. After that results were recorded with natural infection at 15, 30, and 50 days after the second spray treatment in the two successive seasons (2019/2020 and 2020/2021).

##### Determination of Chocolate Spot Disease Severity

The disease severity was recorded after 2, 3 and 5 days of inoculation using the scale (0–9) using the following equation:

Disease severity % = Σ(n × v)/9N × 100(1)

whereas: n = number of plants in every grade, v = numerical grade, N = total number of examined plants, and 9 = maximum disease grade.

### 2.2. Biochemical Analysis

Antioxidant activity of some enzymes performed on the tested inducers best concentration (5 mM for SA, 3 mM for both OA and NA and 3.2 mM BA) which were noticiable in disease severity results. Treated and untreated samples were taken before spraying and 6, 12, 24, and 48 h after spraying. A known weight of *vicia faba* leaves which was extracted in 10 mL of 100 mM phosphate buffer (pH 6.8) and kept at 4 °C overnight. The extract was centrifuged at 5000 rpm for ten minutes and reserved to assay the activities of enzymes [37].

#### 2.2.1. Peroxidase (POX) Assay

The POX activity was assayed according to [38]. Aliquot of 0.2 mL plant enzyme extract was reacted with 5.8 mL of phosphate buffer (50 mM; pH 7.0), 2.0 mL pyrogallol (20 mM) and 2.0 mL hydrogen peroxide (20 mM). The increase in absorbance was determined within 60 s against a reagent without enzyme at 470 nm using a spectrophotometer. The amount of crude enzyme that converts one mM of hydrogen peroxide in one minute at room temperature equals one unit of enzyme activity.

#### 2.2.2. Polyphenol Oxidase (PPO) Assay

The PPO activity was assayed according to Atrooz [39]. A volume of 2.0 mL extract of plant enzyme was reacted with 1.2 mL of phosphate buffer (pH 6.8) and 0.6 mL catechol (2%). The blank tube has only the substrate and the buffer. Thenafter, all samples incubated for 5 min. and the reaction stopped by adding 1 mL of H_2_SO_4_ and the optical density was read at wavelength 430 nm by a spectrophotometer at intervals of 20 min for 100 min. The activity of PPO was expressed as the change in the absorbance of the mixture every 0.5 min. period.

#### 2.2.3. B-1, 3 Glucanase Assay

The method of Abeles and Forrence [40] was used to determine B-1, 3 glucanase activity. Laminarin was used as substrate and dinitro salicylic acid as reagent to measure the reducing sugars. Plant enzyme extract (0.5 mL) was added to 0.5 mL of 0.05 M potassium acetate buffer (pH 5) containing 2% Laminarin. The mixture was incubated for 60 min at 50 °C. The reaction was stopped by adding one ml of dinitrosalicylic acid reagent and heating the tubes for 5 min at 100 °C. The tubes were cooled and 3 mL of distilled water were added before assay. The optical density was adjusted at 500 nm. B-1, 3 glucanase activity was expressed as mM glucose equivalent released gram fresh weight tissues/60 min.

#### 2.2.4. Chitinase Assay

Twenty five grams of chitin was milled, suspended in 250 mL of 85% phosphoric acid (H_3_PO_4_) and stored at 4 °C for 24 h, then blended in 2 L of distilled water using a warning blender and the suspension was centrifuged. This washing procedure was repeated twice. The colloidal chitin suspension in the final wash was adjusted to pH 7.0 with 1 N NaOH, separated by centrifugation and the pelted colloidal chitin was store at 4 °C. The determination was carried out according to the method of [41]. One mL of 1% colloidal chitin in 0.05 M citrate phosphate buffer (pH 6.6) in a test tube, then one ml of enzyme extract was added and mixed by shaking. Tubes were kept in a water bath at 37 °C for 60 min, then cooled and centrifuged before assaying. Reducing sugar was determined by adding 1 mL of supernatant with 1 mL of dintrosalicylic acid and 3 mL distilled water in the test tubes and boiled in water bath for 5 min and then cooled, then determined at 540 nm. Chitinase activity was expressed as mM N-acetyl glucose amine equivalent released gram fresh weight tissue/60 min.

#### 2.2.5. Protein Profile

The electrophoretic protein banding pattern of faba bean leaves. 0.2 g were extracted with 1 mL of protein bufferand kept in the freezer overnight and then vortexed for 15 s and centrifuged at 5000 rpm at 4 °C for 15 min. Then, sodium dodecyl sulfate-polyacrylamide gel electrophoresis (SDS–PAGE) was performed [42], The molecular weight of the isolated proteins was estimatedusing standard molecular weight markers (standard protein markers, 35–320 kDa; Sigma, St. Louis, MO, USA). The protein bands were stained with silver nitrate following the method described by Sammons et al. [43].

### 2.3. Anatomical Studies

Samples were prepared for anatomical investigation according to the method proposed by Nassar and El-Sahhar [44]. One a square centimeter of the terminal leaflet was removed, and it was dehydrated in a succession of solutions with ethyl alcohol concentrations ranging from 50% to 100%. The samples were then embedded in paraffin wax (the melting point of paraffin wax range is 58–62 °C using xylol as a solvent). Sections were cut at a thickness of 15 microns using a rotary microtome and then mounted on slides using egg albumin as an adhesive agent. The slides were subjected to a declining sequence of ethyl alcohol solutions ranging from 100% to 50% ethyl alcohol concentrations. The anatomical characters (Each value represents five sections with five readings each) of faba been leaflet including the upper epidermal layer (µm), lower epidermal layer (µm), palisade tissue thickness (µm), spongy tissue thickness (µm), length of the vascular bundle (µm), and width of the vascular bundle (µm) While in the anatomical study, the percentage (%) was calculated to show the increase or decrease attributed to the control.

### 2.4. Statistical Analysis

A significant differences test among means of five replicates was performed at a signifi cance level of *p* < 0.05 using the LSD (Least Significant Difference) test [45] using the SPSS software. However, under open field conditions, three replicates were used to compare mean differences.

## 3. Results

### 3.1. Severity of Chocolate Spot Disease

**Figure 1:**
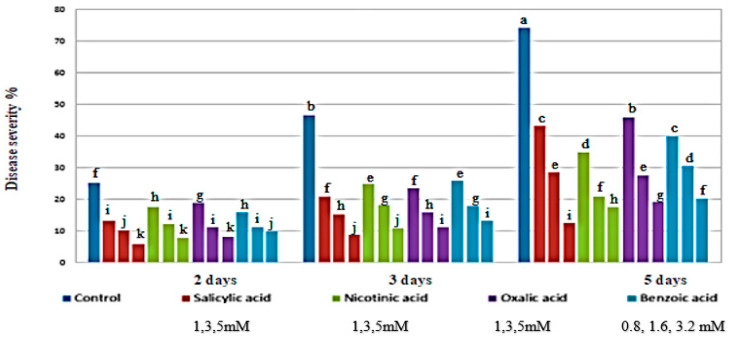
Effect of spraying faba bean plants with varying concentrations of organic acids on the severity of chocolate spot disease under greenhouse conditions. Letters a to k represent significant levels (*p* < 0.05), i.e., a treatment with the letter “a” is significantly different from “b”, and “b” is significantly different from “c”, and so forth. If two treatments have the same letter, they are not significantly different from each other.

Four abiotic inducers, including SA, NA, OA, and BA were investigated for their effects on the severity of chocolate spot disease on faba bean plants grown in greenhouse conditions (Figure 1).

The presented results indicated that all tested abiotic inducers clearly decreased disease severity. There was a positive correlation between the reduction of disease severity and increments in abiotic inducer concentrations. Plants treated with salicylic, nicotinic, oxalic, and benzoic acids reduced chocolate spot disease severity by 83.2, 76.6, 74.2, and 72.8%, respectively, at the highest used concentration.

### 3.2. Development of Chocolate Spot Disease

**Figure 2:**
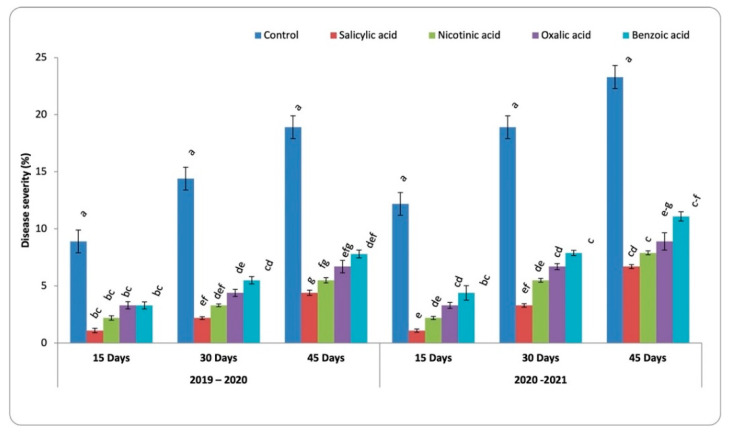
Effect of spraying faba bean with various concentrations of organic acids on the development of chocolate spot disease in field conditions in the two successive seasons. Letters a to k represent significant levels (*p* < 0.05), i.e., a treatment with the letter “a” is significantly different from “b”, and “b” is significantly different from “c”, and so forth. If two treatments have the same letter, they are not significantly different from each other.

Effects of four abiotic inducers (SA, OA, NA, and BA) on chocolate spot disease severity in faba bean, compared to the control, after 15, 30, and 50 days of treatment with inducers under open field conditions are illustrated in Figure 2.

Results showed that spraying faba bean plants with the tested abiotic inducers were able to manage chocolate spot disease using the tested treatments during the two studied seasons. The result indicated that disease severity increased by increasing the plant age. Whereas among abiotic inducers, salicylic acid at a concentration of 5 mM gave the highest reduction through the two seasons by 81.73 and 79.59%, respectively, followed by nicotinic acid at 3 mM concentrations that led to 73.99 and 71.32% reduction, respectively. Benzoic acid at the concentration of 3.2 mM resulted in the lowest reduction in the two seasons by 60.70 and 56.98%, respectively.

### 3.3. Antioxidant Enzymes Activity

**Figure 3:**
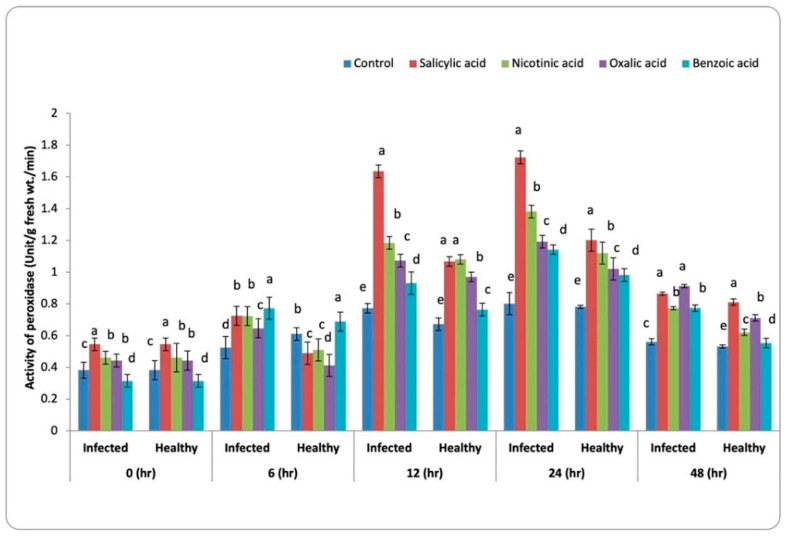
Activity of peroxidase (Unit/g fresh wt./min) in faba bean leaves infested with *B. fabae* plants treated with chemical inducers. LSD 5% for healthy and infected plants at 0, 6, 12, 24, and 48 h are 0.012, 0.012, 0.021, 0.004, 0.018, 0.005, 0.00.016, 0.010, and 0.021, respectively. Letters a to k represent significant levels (*p* < 0.05), i.e., a treatment with the letter “a” is significantly different from “b”, and “b” is significantly different from “c”, and so forth. If two treatments have the same letter, they are not significantly different from each other.

The effect of selected chemical inducers (SA: 5 mM, OA: 3 mM, BA: 3.2 mM, and NA: 3 mM) in addition to the untreated control on the activity of enzymes (Peroxidase, polyphenol oxidase, β-1, 3-glucanase, and chitinase enzymes) were presented in Figure 3. It is worth mentioning that the antioxidant activity was increased with increasing the action time of the content of all tested enzymes till 24 h and then decreased compared to the corresponding control. The healthy faba bean leaves recorded significantly (*p* < 0.05) the lowest value of all antioxidant action compared to the infected plants with *B. fabae*.

#### 3.3.1. Peroxidase Activity (POX)

The obtained values of peroxidase presented in Figure 3 showed a gradual increase with time intervals 0, 6, 12, 24, and 48 h in chemical inducers treated faba bean plants. Foliar spray of faba bean infested with *Botrytis fabae* with various organic acids resulted in a significant increase (*p* < 0.05) of peroxidase activity compared with the untreated control. The maximum increase in peroxidase activity was recorded after 24 h for all treatments. The most pronounced increase was obtained with SA, followed by NA, OA, and BA. Also, peroxidase activity in healthy plants recorded a reduced value compared with infected plants with time intervals in all treatments.

#### 3.3.2. Polyphenol Oxidase (PPO) Activity

**Figure 4:**
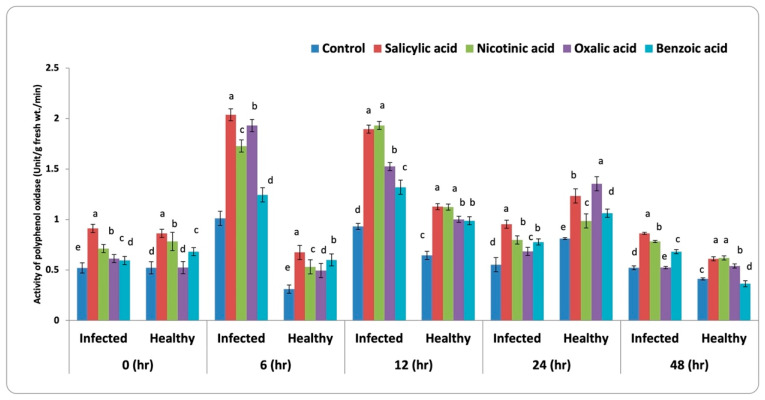
Activity of polyphenol oxidase in faba bean leaves infested with *B. fabae* plants treated with chemical inducers. LSD (5%) for infected and healthy plants at 0, 6, 12, 24, and 48 h are 0.009, 0.008, 0.062, 0.044, 0.099, 0.010, 0.039, 0.011, 0.014, and 0.037, respectively. Letters a to k represent significant levels (*p* < 0.05), i.e., a treatment with the letter “a” is significantly different from “b”, and “b” is significantly different from “c”, and so forth. If two treatments have the same letter, they are not significantly different from each other.

Data presented in Figure 4 indicated that treating faba bean plants infected with *B. fabae* as a foliar treatment with different abiotic inducers resulted in an increase of polyphenol oxidase compared with the untreated infected control.

#### 3.3.3. β-1,3-Glucanase Activity

**Figure 5:**
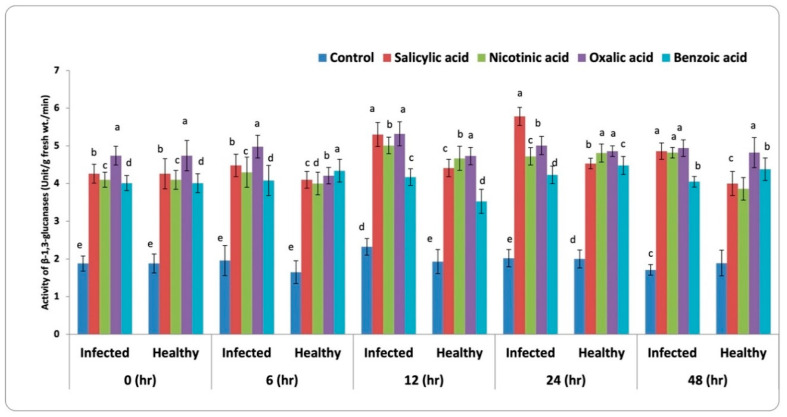
Activity of β-1,3-glucanases in faba bean leaves infested with *B. fabae* treated with chemical inducers. LSD (5%) for infected and healthy plants at 0, 6, 12, 24, and 48 h are 0.096, 0.096, 0.104, 0.061, 0.122, 0.099, 0.083, 0.012, 0.011, and 0.060, respectively. Letters a to k represent significant levels (*p* < 0.05), i.e., a treatment with the letter “a” is significantly different from “b”, and “b” is significantly different from “c”, and so forth. If two treatments have the same letter, they are not significantly different from each other.

Data presented in Figure 5 showed a significant increase in the β-1, 3-glucanase activity in all treatments during the examination periods compared to the control in both infected and healthy plants. Some treatments recorded the highest increase in β-1, 3-glucanase activity after 12 h, while other treatments recorded the highest values after 24 h. For infected faba bean plants, OA and SA caused the maximum increase in β-1, 3-glucanase after 12 h, followed by BA. Meanwhile, SA recorded the maximum increase after 24 h, followed by OA then BA came in the third order. The increase of β-1, 3-glucanase activity was more than two-fold of the untreated control. In the case of healthy faba bean plants, all chemical inducers produced the highest increase in β-1, 3-glucanase activity after 24 h, where OA came in the first order, followed by NA, then SA, and BA treatments. After that, the activity reduced slightly at 48 h but was still higher than the untreated control. 

#### 3.3.4. Chitinase Activity

**Figure 6:**
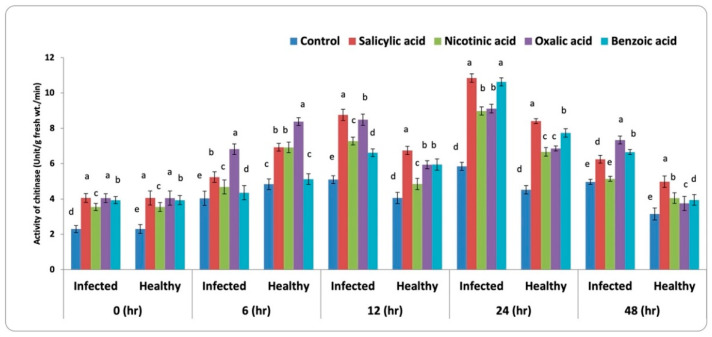
Activity of chitinase in leaves of faba bean infested with *B. fabae* plants treated with chemical inducers. LSD 5% for infected and healthy plants at 0, 6, 12, 24, and 48 h are 0.011, 0.011, 0.120, 0.101, 0.103, 0.066, 0.079, 0.086, 0.105, and 0.049, respectively. Letters a to k represent significant levels (*p* < 0.05), i.e., a treatment with the letter “a” is significantly different from “b”, and “b” is significantly different from “c”, and so forth. If two treatments have the same letter, they are not significantly different from each other.

A perusal of data in Figure 6 showed that the infected and healthy faba bean foliar treated with different abiotic inducers was associated with increased chitinase activity compared with the untreated corresponding control. All treatments increased significantly (*p* < 0.05) chitinase activity. Salicylic acid recorded the maximum increase in this concern after 24 h of both infected and healthy plants, followed by benzoic and oxalic acids, respectively. Meanwhile, nicotinic acid recorded the lowest value compared to untreated plants (control).

### 3.4. Protein Electrophoretic Banding Patterns

A banding pattern of the soluble proteins in infected and healthy leaves of faba bean that belongs to the cultivar Giza 429 infected with *B. fabae* and treated with selected chemical inducers. The inducers included salicylic acid (5 mM), oxalic acid (3 mM), benzoic acid (3.2 mM) and nicotinic acid (3 mM), via SDS-PAGE as presented in Table 1. A total of 27 polypeptides of faba bean leaves displayed heterogeneity compared to control. The infected and treated plants with some chemical inducers with molecular weights (MWs) ranged from 35 to 320 kDa after 24 and 48 h of application and 43 kDa in response to oxalic and benzoic acids only after 24 h from the application. Meanwhile, after 48 h from the application, data showed a new protein polypeptide bands appeared at Mwt. of 172, 104, 59, 44, and 35 kDa due to all organic acid treatments (tested chemical inducers) compared to the control plants. The number if protein bands increased due to the salicylic, nicotinic, oxalic, and benzoic acid treatments, which recorded the numbers of 8, 9, 16, and 14 bands after 24 h from the application. Meanwhile, the protein bands 11, 11, 13, and 11 were recorded after 48 h from the application. The most pronounced increases were observed in response to oxalic acid treatment, which recorded 16 bands after 24 h and 13 after 48 h of application, followed by benzoic acid, which recorded 14 and 11 bands, respectively, after 24 and 48 h. It is evident from the obtained data that all the treatments of faba bean plants with organic acids cause the appearance of polypeptide protein bands with Mwt. of 59 and 51 kDa.

**Figure 7:**
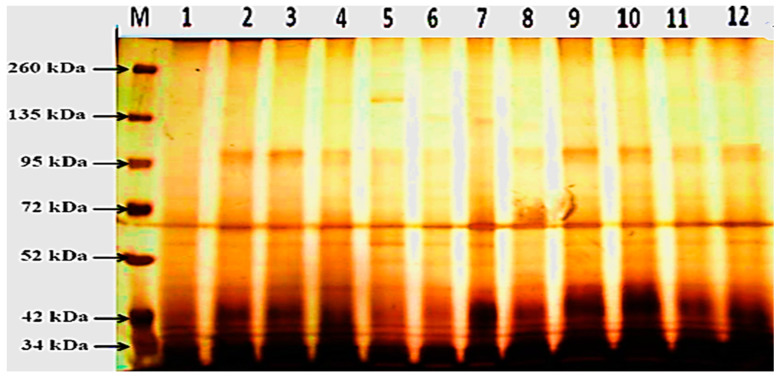
Separation of soluble proteins (SDS-PAGE) in faba bean plant leaves treated with chemical inducers against *B. fabae*.

Data in Figure 7 showed the appearance of a new band at Mwt. of 176, 85, 65, 59, 51, and 41 in response to NA, OA, and BA, and also 172, 130, and 77 kDa. 

The infected faba bean plants with *Botrytis fabae* induced a *de novo* synthesis of new polypeptide bands that appeared at molecular weights 297, 104, 66, 59, and 43 kDa after 24 h of application and also at 104, 59, and 35 kDa after 48 h of application. At the same time, there is a disappearance of 3 protein bands with molecular weight 98, 78, and 66 after 24 h of application, and after 48 h, only one band (OA treatment) was absent at Mwt 137 kDa.

Lane M: Marker, (1) Healthy control after 24 h from the application, (2) Infected control after 24 h from application, (3) Salicylic acid after 24 h from the application, (4) Nicotinic acid after 24 h from the application, (5) Oxalic acid after 24 h from the application, (6) Benzoic acid after 24 h from application, (7) Healthy control after 48 h from the application, (8) Infected control after 48 h from the application, (9) Salicylic acid after 48 h from the application, (10) Nicotinic acid after 48 h from the application, (11) Oxalic acid after 48 h from application and (12) Benzoic acid after 48 h from the application.

### 3.5. Anatomical Characteristics

**Figure 8:**
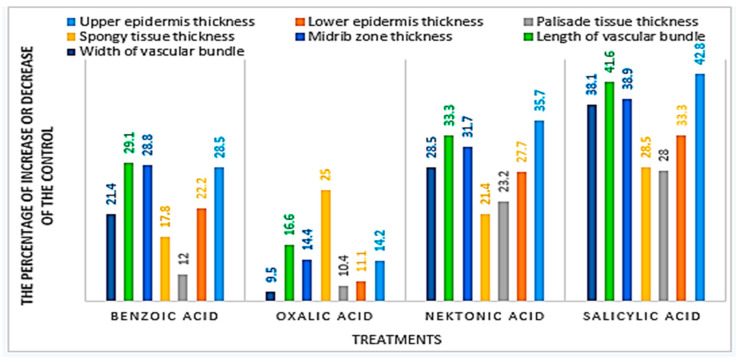
Effect of foliar application with chemical inducers on the anatomical characteristics of faba bean (*Vicia faba* L.) terminal leaflet under chocolate spot disease stress.

**Figure 9:**
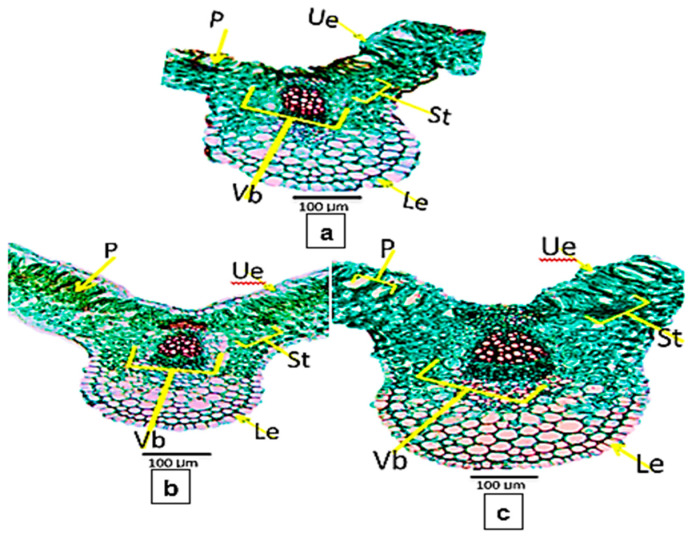
Transverse-sections through blade of the terminal leaflets of faba bean (*Vicia faba* L.) under chocolate spot disease: (**a**) Untreated plant (Control), (**b**) Plant treated with benzoic acid and (**c**) Plant treated with salicylic acid. Abbreviations: Le = Lower epidermis; P = Palisade tissue; St = Spongy tissue Ue = Upper epidermis and Vb = Vascular bundle.

Microscopically counts and measurements of specific histological characteristics in transverse-sections through the blade of mature leaflet of faba been plant, benzoic, and salicylic acids presented in Figure 8 and Figure 9a–c. The SA (Figure 9c) recorded the highest value with an increase in leaf thickness by (+30.1%) more than the control (Figure 9a). Such increase in leaf thickness corresponds with the enhancement recorded on the upper and lower epidermal layer thickness as well as palisade and spongy tissues by +42.8, +33.3, +28.0 and +28.5%, respectively more than the control. Such response also resulted in a clear appearance and arrangement of spongy and palisade tissue parenchymatous cells as compared to the control. However, foliar applications with BA (Figure 9b) recorded the lowest value with increased leaf thickness by (+15.8%) more than the control. Such increase in leaf thickness is related to the increment recorded in the upper and lower epidermal layer thickness as well as palisade and spongy tissues by (+28.5%, +22.2%, +12.0%, and +17.8%) respectively, more than the control.

This effect is associated with vast intercellular spaces between both palisade and spongy tissues parenchymatous cells. Furthermore, parenchymatous cells of upper and lower epidermal layers, as well as palisade and spongy tissues, are bigger in size and rounded in shape under treatment with SA. Clearly, Figure 8 and Figure 9a–c revealed that foliar application with SA recorded the highest value with increased thickness of the midrib zone by (+38.9%) more than the control. Such an increase in midrib zone thickness corresponds with the enhancement recorded on the length and width of the vascular bundle as well as the diameter of xylem vessels by +29.1, +21.4, and +45.4%, respectively, more than the control.

In addition, the area occupied by collenchymatous cells behind the main vascular bundle is occupied by larger sizes and more layers of collenchymatous cells. Such effect corresponds with clear development and differentiation of the main vascular bundle’s elements, especially xylem vessel elements. Also, metaxylem vessels also changed from a rounded shape, as recorded in the control, to an elongated shape in the treated plants. This response is mainly due to the increment that occurred in the area occupied by vascular bundle elements, which appeared in the area coupled by xylem vessels, cambium, and phloem as compared to the untreated plants.

## 4. Discussion

### 4.1. Severity of Chocolate Spot Disease

The greenhouse experimental results indicated that pre-treated faba bean plants with abiotic inducers foliar treatment resulted in a significant reduction in disease severity of *B. fabae* compared with untreated control. Using bioagent isolates revealed antagonistic activity against *B*. *fabae* due to the production of protease, lipase, IAA, and ammonia. Moreover, bioagents release tricalcium phosphate (TCP) which promote multiple plant growth characteristics [46]. Abiotic inducers; SA, NA, OA, and BA gave a reduction of 83.2, 76.6, 74.2, and 72.8% at the highest concentration of each treatment. Among all foliar treatments, SA spray had the highest reduction of 83.2% at 5 mM of 5-day treatment. A second highest reduction of 80.9% was observed at a 3-day treatment with SA with 5 mM concentration. This was followed by NA application that reduced disease severity by 76.7% and 76.6% in 3-day and 5-day, respectively. The third highest reduction in severity was observed with OA application that reduced the severity by 75.8% and 74.2% in 3-day and 5-day treatments, respectively. While the fourth highest reduction was obtained with BA application at 5 mM, which reduced the disease severity by 72.8% and 71.5% in 5-day and 3-day treatments, respectively.

This agrees with Metwaly [47], who studied the evaluation of some chemical inducers, i.e., ascorbic, citric, salicylic acids, and calcium chloride (as a nutrient salt) to control faba bean chocolate spot disease. The study concluded that all tested organic acids significantly reduced the in vitro mycelial growth of the pathogenic fungus (*B. fabae*), and complete growth inhibition was recorded when 2500 ppm concentration was applied [47]. SA was the most effective one, followed by ascorbic acid. Plant treatment with organic acids and calcium chloride under greenhouse and/or field conditions led to a significant effect on controlling the disease and increasing phenols content as well as the activity of oxidative enzymes [6]. The results also agreed with the results of salicylic, citric, ascorbic, and oxalic acids as effective chemical inducers.

Under field conditions, plants treated with SA significantly reduced the disease severity in comparison with the control treatment. SA was highly efficient in controlling the fungal disease compared to other treatments that provided limited partial protection. In this regard, Thakur et al. [48] explained that chemical inducers are elicitors’ compounds that activate plant chemical defense. Activation of a variety of biosynthetic pathways was observed in treated plants, which depended upon the used compound. Commonly, the studied elicitors include SA, methyl salicylate, benzothiadiazole, BA, and chitosan, with a key role in phenolic acid production as well as activation of several plant enzymes involved in defense mechanisms. Also, Zian et al. [49] observed that using SA led to a significant decrease in the root rot and wilt diseases of lupine. They observed that the SA leads to an increase in the activity of chitinase, β-1, 3-glucanase, peroxidase, and polyphenoloxidase. Morever, the induced resistance caused by some abiotic (BTH) and/or biotic inducers might provide a practical, eco-friendly management approach to chocolate spot disease when they are combined with suitable agronomic practices [50]. In their study, applying 0.3 and 0.5 mM benzothiadiazole as the foliar treatment significantly reduced the severity of chocolate spot disease.

### 4.2. Antioxidant Enzymes Activity

The antioxidant activities by enzymes, such as peroxidase, polyphenol oxidase, β-1, 3-glucanase, and chitinase in the leaves of faba bean plants increased significantly under biotic stress (Figure 3a–d). In this regard, Gholami et al. [51] reported that these antioxidant enzymes were thought to be part of a preservation system that reduced oxidative damage caused by the increased formation of reactive oxygen species (ROS) due to biotic and abiotic stress. Higher content of H_2_O_2_ is detoxified at stress conditions through catalase and glutathione peroxidase [52].

Interestingly, the antioxidant system in plants includes enzymes that scavenge ROS, such as ascorbate peroxidase, catalase, peroxidase, and superoxide dismutase [26]. Sairam and Srivastava [53] determined that plants having higher levels of either inducible or constitutive enzymes showed to be more resistant to oxidative harmful effect. OA appears to have a biphasic effect on ROS metabolism since it suppresses ROS buildup at first and activates their generation at later phases of pathogen infection [25]. Application of bioagents was characterized by the expression of cyclolipopeptides (cLP) genes, encoding for induced resistance factors, as well as the production of lipopeptides, indoleacetic acid, siderophores, hydrocyanic acid, and extracellular enzymes such as amylase, protease, pectinase, and cellulase [54].

Pretreatment of faba bean plant (infected and healthy plants) with the studied natural organic acids improved stress tolerance by increasing the tested antioxidant enzyme activities compared to control. Furthermore, Sharma and Dubey [55] observed that a viable protection against oxidative damage was produced by this antioxidant system. This enhances active oxygen species lifetime in the cellular environment. The defense mechanism of plant β-1, 3-glucanases is directly through suppressing ROS buildup and/or hydrolyzing the fungal pathogen cell walls along with chitinase isozymes. Laboratory studies showed that fungal pathogens were directly affected by β-1, 3-glucanases through deteriorating b-1, 3/1, 6-glucans. Also the chitinases produced their effects via the C1 and C4 bond that are consecutive N-acetylglucosamines of chitins in the cell walls of fungi [56]. The plants treated with salicylic acid increased the activity of enzymes such as catalase, peroxidase, superoxide dismutase, ascorbate peroxidase, and glutathione reductase. [57]. Peroxidase belongs to oxido-reductase enzymes and contributes to oxidation-reduction reactions.

Concerning the effect of benzoic acid on the antioxidant enzyme system, a similar result was obtained by Zhang et al. [58] on tomato seedlings and Amist and Singh [59] on wheat seedlings, who found that the increasing antioxidant enzymes seem to protect plants from oxidative stress. Hassanein et al. [60] found that 100 ppm of nicotinamide (either soaking or spraying) increased the content of antioxidant enzymes (Superoxide dismutase, peroxidase, and catalase) and decreased lipid peroxidation of *Zea mays* plants. Also, in this regard exogenous application of methyl jasmonate increased the activity of antioxidant enzymes, improved photosynthetic pigments and PSII efficiency. This resulted in enhanced growth of pea plants under Cd stress. The improved traits included increments in the fresh and dry weights of shoots and roots. Thus, the mitigating effect of methyl jasmonate was due to its role in cellular redox balance and photosynthetic mechanism of plants under Cd stress conditions [54].

### 4.3. Changes in Protein Electrophoretic Patterns

The data clearly demonstrated that, in general, after 24 h from the application, there was an absence of some polypeptide bands at molecular weights 117 in response to salicylic acid and 98, 78, and 66 kDa in response to nicotinic acid treatments. In this respect, the disappearance of protein bands and also the synthesis of a new group of soluble proteins were observed by many authors due to biotic and abiotic stress. The absence of some bands may result from inherited effects of infection with *B. fabea*, which explains the basis of the mutational event on the regulatory genes that prevent or attenuate transcription [61].

It is obvious from the obtained results (Figure 4 and Table 1) that all treatments of faba bean plant with organic acids cause the appearance of the polypeptide protein band with Mwt. Of 59 and 51 kDa. In this regard, Spreitzer and Savucci [62] discovered that the 51 kDa band, that might be associated with Ribulose-1, 5-bisphosphate carboxylase activase (Rubisco activase), was elevated. By promoting the separation of firmly bound sugar-phosphates from Rubisco in an ATP-dependent mechanism, this enzyme could modify the Ribulose-1 activity, 5-bisphosphate carboxylase/oxygenase (Rubisco), a crucial enzyme involved in the initiation of photosynthetic and photorespiratory carbon metabolic processes. Enhancing Rubisco’s capability has important implications for plant productivity and resource use efficiency [63]. Moreover, these total proteins have osmoprotectant functions and protective effects on cellular structures [64].

Amino acids are primary metabolites that play essential roles in plant immunity against many pathogens. The variation in plant tissues amino acid quantity may determine the chance of environment for the pathogenic attackers like fungi, bacteria, and viruses. This finally strengthens plant defense to resist pathogenic attack effectively or surrender before vigorous infection [65]. Morphological and structural barriers, chemical substances, proteins, and enzymes are all examples of biotic stress protection mechanisms. By preserving products and giving them strength and stiffness, they confer tolerance or resistance to biotic stressors. In addition, amino acids serve an important function in stress response and secondary metabolism in plants [66]. For scavenging ROS resulting from biotic stress (infection with *B. fabae*), the plant has a protective system against the devastating oxidative reaction. The protective system includes osmoprotectants (total soluble protein) and antioxidative enzymes, which are known to play a crucial role in defense mechanisms, and all of them are proteins and can appear as protein bands. In this regard, Ramadan et al. [67] on flax plant found that benzoic acid increased total soluble protein and free amino acids. Also, Mahgoob and Talaat [68] found that foliar application of nicotinic acid at 50 mg/L significantly increased total protein contents of rose geranium.

The appearance of *de novo* synthesis of new polypeptides band and/or increase the band density 77, 59, 51 and 41 may represent an antioxidant enzyme (chitinase and β-1, 3-glucanase) which is considered as defense proteins to protect the plant from pathogens. In ripe cherimoya fruits (*Annona cherimola* Mill.), Goni et al. [69] found a highly expressed constitutive chitinase (at 27 kDa), as well as the creation of a unique acidic chitinase (at 26 kDa) and a 1, 3-glucanase (51 kDa). Likewise, a new basic chitinase at 33 kDa was observed in the post-ripening stage of fruits that were stimulated, as well as another basic constitutive β-1, 3-glucanase at the molecular weight of 76 kDa. In vitro, extracts of these acidic and basic proteins suppressed the growth of *B. cinerea*, a necrotrophic fungal pathogen in grape leaves. At the berries’ harvest stage, defense proteins were dominant, particularly various chitinase and β-1, 3-glucanase isoforms that support fruit ripening. Also, Shukry [70] explained that the hydrin is defined at the protein band with Mwt. of 40 kDa. These proteins play a preventative role during water stress through their function as ion traps in dehydrating cells and insulating ions as their concentration increases.

### 4.4. Anatomical Characteristics

The present experimental results (Figure 8 and Figure 9a–c) clear that foliar application with salicylic acid at 3 mM on faba been plant led to the highest increase in the thickness of the upper, lower epidermis, leaf blade thickness, palisade, or spongy tissue midrib zone, length, and width of the vascular bundle and. Also, foliar application with salicylic acid produced great increase in the most mentioned layers more than the control. Although the foliar application with all treatments led to a slight increase in the thickness of the examined layers was recorded especially by benzoic acid, compared to the untreated plants. In this regard, Ali et al. [71] on maize (*Zea mays*, L.) reported that treatment with citric acid led to increasing the upper and lower epidermal layers, length of the vascular bundle, and mesophyllic tissue, while the width of vascular bundle was similar to the control. Concerning foliar application with salicylic acid, the results showed an increase of thickness in both palisade and spongy tissues; these results are in agreement with Gomaa et al. [18], who observed that foliar spraying of the cultivar ‘Giza 2, Egyptian lupine, with salicylic acid increased the thickness of leaflets lamina and midvein. This is due to an increase in the thickness of spongy and palisade tissues as well as to increase in the midvein bundle size. Some investigators confirmed the present findings using salicylic acid on other field crop plants, for instance, Cárcamo et al. [16] on *Zea mays*, L., Nour, et al. [17] on bean, Gomaa et al. [18] on *Lupinus termis* L. and Khalil, et al. [72] on pea plants. They found that salicylic acid application increased the thickness of the midvein and lamina of the studied plant leaves. Also, Maddah et al. [73] found that spraying salicylic acid with 0.1 mM increased the stomata number, but watering plants with 1.5 mM of salicylic acid damages the parenchyma tissues in leaves, the tissue of sclerenchyma in stems, and xylem in the root, the exogenous spraying of SA might alleviate the inhibitory impact of salinity stress on the growth, physiological and anatomical features, and the productivity of cowpea plants El-Taher et al. [74].

## 5. Conclusions

This study investigates the positive role of organic acids in alleviating abiotic stress conditions, which that can be beneficial for improving the performance of faba bean plants to increase the productivity of land area units. It was found that the uses of these phenolic materials not only eliminate the pathogens but also improve the characteristics of the plant to resist diseases (Chocolate spot disease). Whereas among abiotic inducers, salicylic acid at the concentration (5 mM) gave the highest reduction (control chocolate spot disease) through the two seasons respectively. This research will assist the researcher in uncovering crucial regions of secondary metabolites defense causative agents of plants against abiotic stress that many researchers have yet to investigate.

## Figures and Tables

**Table 1:** Separation of soluble proteins (SDS-PAGE) in faba bean leaves treated with various chemical inducers against *B. fabae*.

Band No.	Marker	After 24 h from Application						After 48 h from Application					
	Molecular Weight (kDa)	Control		SA	NA	OA	BA	Control		SA	NA	OA	BA
		Healthy	Infected					Healthy	Infected				
1	320	+	+	+	+	+	+	+	+	+	+	+	+
2	301	−	−	+	−	−	−	−	−	−	−	−	−
3	399	−	−	−	−	+	−	−	−	−	−	−	−
4	297	−	+	−	−	−	−	−	−	−	−	−	−
5	176	−	−	−	+	+	+	−	−	−	−	−	−
6	172	−	−	+	−	+	+	−	−	+	+	+	+
7	137	−	−	−	−	−	−	+	−	+	+	−	+
8	130	−	−	−	−	+	+	+	+	−	−	+	−
9	117	+	+	−	−	+	+	−	−	−	−	−	−
10	116	−	−	−	−	−	−	−	−	−	−	−	+
11	104	−	+	+	+	−	−	−	+	+	+	+	+
12	98	+	−	−	−	+	−	−	−	−	−	−	+
13	85	−	−	−	+	+	+	−	−	−	−	+	−
14	84	−	−	−	−	−	+	+	+	+	−	−	−
15	78	+	−	−	−	−	−	−	−	−	+	+	−
16	76	−	−	−	−	+	+	−	−	−	−	−	−
17	66	+	−	−	−	−	−	+	+	−	+	+	+
18	65	−	+	+	+	+	+	−	−	+	−	−	−
19	62	−	−	−	−	+	−	−	−	−	+	−	−
20	59	−	−	−	+	+	+	−	+	+	+	+	+
21	51	−	−	+	+	+	+	+	+	+	+	+	+
22	44	−	−	−	−	−	−	−	−	+	+	+	−
23	43	−	+	+	−	+	+	+	+	−	−	−	−
24	42	−	−	−	−	−	−	−	−	+	−	+	−
25	41	−	−	−	+	+	+	+	+	−	−	−	+
26	40	−	+	+	+	+	+	−	−	−	−	+	−
27	35	−	−	−	−	−	−	−	+	+	+	+	+
Total bands		5	7	8	9	16	14	8	10	11	11	13	11

+ = Presence of band and − = Absence of band.

## Data Availability

Data will be made available with a reasonable request to the corresponding authors.
